# Low dosage combination treatment with metformin and simvastatin inhibits obesity-promoted pancreatic cancer development in male KrasG12D mice

**DOI:** 10.1038/s41598-023-43498-9

**Published:** 2023-09-26

**Authors:** Yaroslav Teper, Linda Ye, Richard T. Waldron, Aurelia Lugea, Xiaoying Sun, James Sinnett-Smith, Oscar J. Hines, Stephen J. Pandol, Enrique Rozengurt, Guido Eibl

**Affiliations:** 1 Department of Surgery, David Geffen School of Medicine, University of California, Los Angeles, CA USA; 2 Pancreatic Research Group, Department of Medicine, Cedars-Sinai Medical Center, Los Angeles, CA USA; 3 Department of Medicine, David Geffen School of Medicine, University of California, Los Angeles, CA USA

**Keywords:** Pancreatic cancer, Cancer prevention

## Abstract

Pancreatic ductal adenocarcinoma (PDAC), a highly lethal disease with limited therapeutic options, may benefit from repurposing of FDA-approved drugs in preventive or interceptive strategies in high-risk populations. Previous animal studies demonstrated that the use of metformin and statins as single agents at relatively high doses restrained PDAC development. Here, four-week-old mice expressing KrasG12D in all pancreatic lineages (KC mice) and fed an obesogenic high fat, high calorie diet that promotes early PDAC development were randomized onto low dosage metformin, simvastatin, or both drugs in combination administered orally. Dual treatment attenuated weight gain, fibro-inflammation, and development of advanced PDAC precursor lesions (pancreatic intraepithelial neoplasia [PanIN]-3) in male KC mice, without significant effect in females or when administered individually. Dual-treated KC mice had reduced proliferation of PanIN cells and decreased transcriptional activity of the Hippo effectors, YAP and TAZ, which are important regulators of PDAC development. Metformin and simvastatin also synergistically inhibited colony formation of pancreatic cancer cells in vitro. Together, our data demonstrated that a combination of low doses of metformin and simvastatin inhibits PDAC development and imply that both drugs are promising agents for being tested in clinical trials for preventing pancreatic cancer progression.

## Introduction

Pancreatic cancer, of which pancreatic ductal adenocarcinoma is the most common histologic subtype, continues to be a lethal disease despite incremental advances in surgery, imaging, and chemotherapeutic regimens. According to the American Cancer Society, an estimated 33,130 men and 30,920 women in the United States will be newly diagnosed with pancreatic cancer in 2023^1^. The incidence of pancreatic cancer has risen steadily over the past decade with an alarming 46% and 38% increase in expected new cases from 2013^2^ to 2023^1^ in men and women, respectively. Similarly, in the European Union (plus United Kingdom) the number of new pancreatic cancer cases was 59,000 in 1990 and is projected to be 147,000 in 2039^3^, an almost 150% increase. This dramatic rise in pancreatic cancer is thought to be caused, at least in part, by a substantial increase in prevalent risk factors, in particular obesity and type 2 diabetes^4^. In addition, an estimated 26,620 men and 23,930 women in the United States will die from pancreatic cancer in 2023, rendering this disease the third most common cause of cancer-related deaths in men and women combined, approaching colorectal cancer^1^. The five‐year relative survival for patients with pancreatic cancer for all stages (data from 2012 to 2018) stands at 12%^1^. Although the survival doubled compared to 6% a decade prior^2^, it is still disappointingly and unacceptably low and has the worst survival rate among the most common human cancers. It is evident that substantial progress still needs to be made to improve the outcome of patients with pancreatic cancer. Besides from the discovery of new efficacious and targeted cytotoxic drugs, major advancements will need to come from a detailed molecular understanding of risk factors for pancreatic cancer leading to the development of preventive and/or interceptive strategies^5^.

The re-purposing of FDA-approved drugs thereby constitutes a promising avenue for pancreatic cancer prevention/interception. In this regard, metformin, the most widely prescribed drug for the prevention and treatment of type 2 diabetes mellitus worldwide, and statins, approved for lowering blood lipid levels, have been shown in large epidemiological studies to be associated with a lower risk of pancreatic cancer^6–10^. We have previously demonstrated that oral administration of either metformin (in drinking water) or simvastatin (in diet) significantly reduced pancreatic fibro-inflammation and cancer development in a genetically engineered mouse model of pancreatic cancer subjected to diet-induced obesity^11,12^. In this model (*KrasG12D;p48-Cre* [KC]) an oncogenic *KrasG12D* mutation is conditionally expressed in all pancreatic cell lineages during embryologic development. These KC mice will develop acinar-to-ductal metaplasia (ADM) and progressive pancreatic intraepithelial neoplasia (PanIN-1 to -3) lesions, recognized pancreatic cancer precursors, after birth, which progress in a small percentage of mice into invasive pancreatic cancer at around 9 months^13^. Diet-induced obesity has been shown to dramatically accelerate the development of invasive pancreatic cancer in this model^14,15^. In our previous studies, the beneficial effects of metformin and simvastatin were seen at clinically relevant but relatively high levels of each compound. Metformin and simvastatin are known to cause clinically relevant side effects in a small subset of patients, most commonly gastrointestinal irritation^16^ and myopathies^17^, respectively. Since these adverse effects at high doses can lead to cessation or interruption of medication use, it is pertinent to study whether lower doses of metformin and simvastatin similarly exhibit anti-cancer effects in preclinical models of pancreatic cancer.

Mechanistically, the direct anti-cancer properties of metformin and simvastatin are thought to be mediated, at least partially, by their inhibitory action on the transcriptional co-activators YAP (Yes-associated protein) and TAZ (transcriptional co-activator with PDZ-binding motif)^18,19^, major downstream effectors of the Hippo pathway, which gained strong interest as a central hub for (pancreatic) cancer development and progression^20–23^. Since metformin and simvastatin regulate and inhibit YAP/TAZ transcriptional activity via different mechanisms^18,19^, we hypothesized that the combination of low doses of metformin and simvastatin attenuates pancreatic cancer development in the KC mouse model with diet-induced obesity, while the administration of each drug alone at low concentration has no effect. Our results show that oral administration of metformin and simvastatin at low concentrations in combination significantly improved pancreatic fibrosis and inflammation and reduced pancreatic cancer development in male, but not female, KC mice with diet-induced obesity, while treatment with either drug alone at these concentrations had no effect.

## Results

### The combination of metformin and simvastatin at low concentrations improves pancreatic fibro-inflammation in male KC mice fed the high fat, high calorie diet

**Figure 1:**
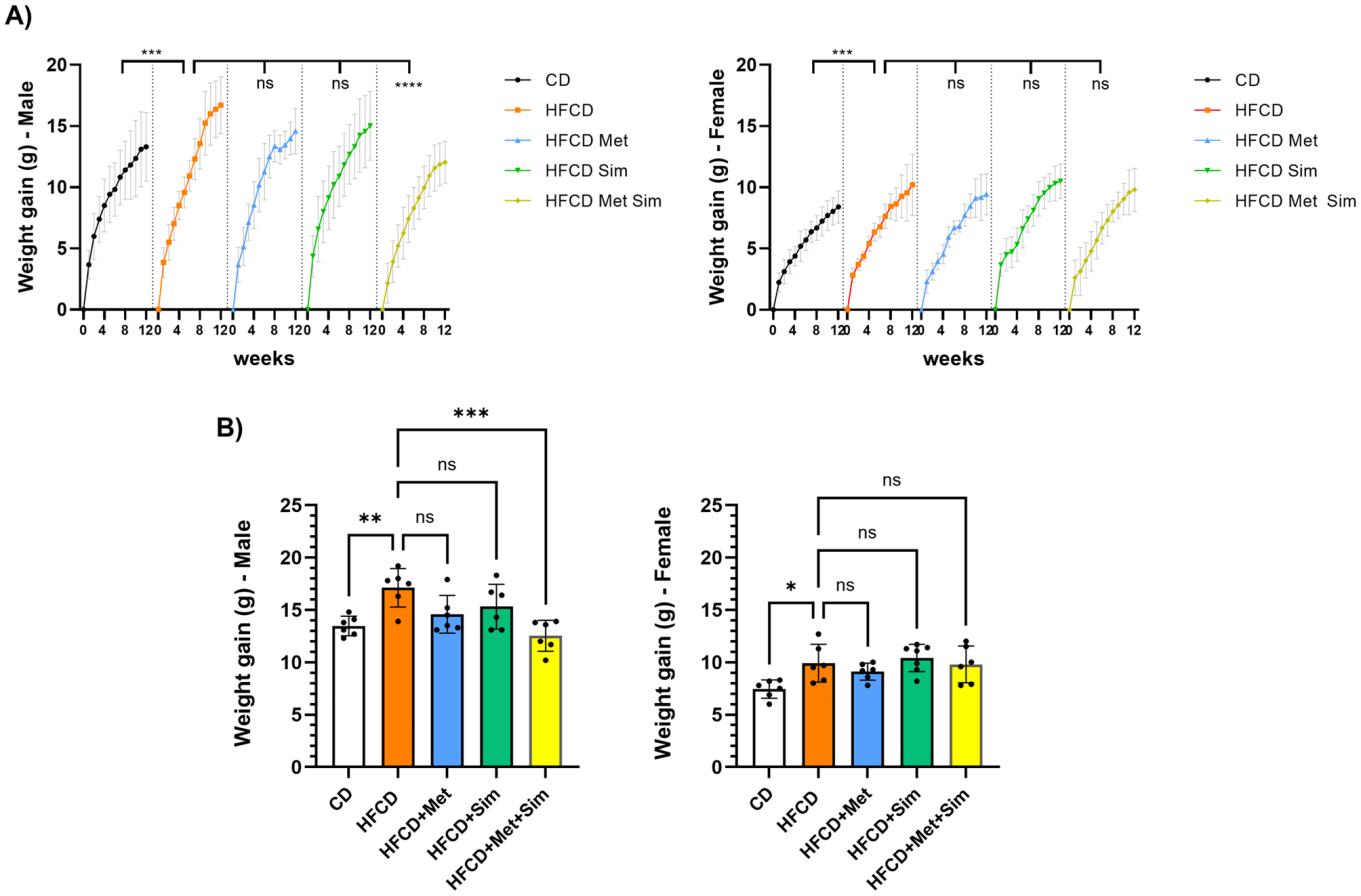
(**A**) Weekly weight gain in male (left panel) and female (right panel) KC mice fed the control diet (CD), high fat, high calorie diet (HFCD), HFCD plus metformin (Met), HFCD plus simvastatin (Sim), or HFCD plus metformin and simvastatin (Met Sim). ***p ≤ 0.001, ns = not significant. (**B)** Weight gain at the end of the study (after 3 months of treatment) in male (left panel) and female (right panel) KC mice fed the CD, HFCD, HFCD + Met, HFCD + Sim, or HFCD + Met + Sim. Data are presented as scatter plots with bars (mean ± SD). ***p ≤ 0.001, **p ≤ 0.01, ns = not significant.

To study the effects of low doses of metformin and simvastatin alone and in combination, male and female KC mice were fed the (i) control diet (CD), (ii) high fat, high calorie diet (HFCD), (iii) HFCD plus metformin (1 mg/ml in drinking water), (iv) HFCD plus simvastatin (50 mg/kg in the diet), or (v) HFCD plus metformin (1 mg/ml) and simvastatin (50 mg/kg) for 3 months. Similar to our previous studies^11,12,14,15^ animals fed the HFCD gained significantly more weight than KC mice fed the CD (Fig. 1A, B). While female and male KC mice after 3 months of feeding the CD gained 7.5 ± 0.9 g and 13.5 ± 0.9 g, respectively, the increases in body weight of female and male KC mice fed the HFCD for 3 months were 9.9 ± 1.8 g and 17.1 ± 1.8 g, respectively (p = 0.03 in females HFCD vs. CD and p = 0.008 in males HFCD vs. CD). Administration of metformin alone or simvastatin alone had no significant effect on weight gain in female and male KC mice on HFCD (Fig. 1A, B). However, male KC mice fed the HFCD with the combination of metformin and simvastatin gained significantly less weight after 3 months of feeding compared to male KC mice fed the HFCD alone (12.5 ± 1.5 g vs. 17.1 ± 1.8 g; *p* < 0.001) (Fig. 1B). In contrast, the combination of metformin and simvastatin had no effect on weight gain in female KC mice compared to HFCD alone.

**Figure 2:**
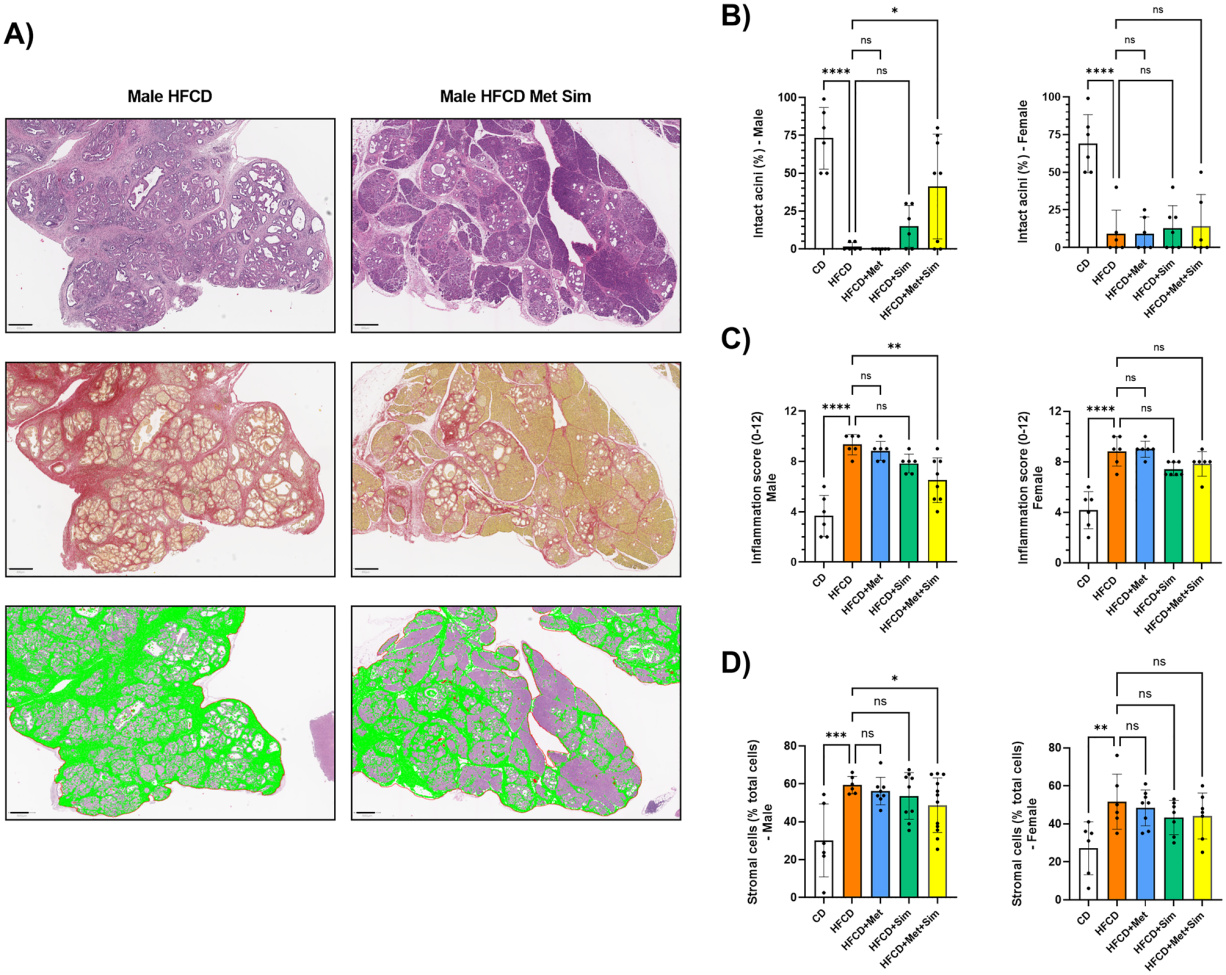
(**A**) Representative microscopic images of the pancreas of male KC mice fed the high fat, high calorie diet (HFCD) (left column) or HFCD plus metformin (Met) and simvastatin (Sim) (right column). In the first row, tissue sections were stained with hematoxylin and eosin (scale bar: 400 µm). In the second row, tissue was stained with Sirius red (scale bar: 400 µm). The third row represents QuPath analysis of stromal cells highlighted in green (scale bar: 500 µm). (**B**) Percentage of intact pancreatic acini, (**C**) pancreatic inflammation score, and (**D**) percentage of stromal cells in male (left panel) and female (right panel) KC mice fed the control diet (CD), HFCD, HFCD + Met, HFCD + Sim, or HFCD + Met + Sim. Data are presented as scatter plots with bars (mean ± SD). ****p ≤ 0.0001, ***p ≤ 0.001, **p ≤ 0.01, *p ≤ 0.05, ns = not significant.

Histologically, the pancreas of female and male KC mice fed the HFCD had marked signs of robust fibro-inflammation (Fig. 2A). Compared to the CD, animals fed the HFCD were characterized by a significant loss of intact pancreatic acinar cells (Fig. 2B), a marker of tissue destruction and neoplastic development, and an increase in the pancreatic inflammation score (Fig. 2C), a composite histological score comprising of semi-quantitative analysis of pancreatic acinar cell loss, inflammatory cell infiltration, and fibrosis^15^. The loss of intact pancreatic acinar cells and the pancreatic inflammation score in female and male KC mice fed the HFCD with metformin alone or simvastatin alone were not significantly different compared with those fed the HFCD. However, the most striking finding in the histological examination was the significant improvement in pancreatic architecture in male KC mice fed the HFCD with the combination of metformin and simvastatin (Fig. 2A–C), as reflected by a greater preservation of intact pancreatic acinar cells and a reduced pancreatic inflammation score (HFCD: 9.3 ± 0.8 vs. HFCD + Met + Sim: 6.5 ± 1.8; p = 0.003). The reduction in fibrosis in male KC mice fed the HFCD with metformin and simvastatin (as compared to HFCD alone) was qualitatively confirmed by Sirius Red staining (Fig. 2A) demonstrating a marked decrease in collagen deposition. In addition to the reduced area of fibrosis and collagen deposition, the administration of HFCD plus metformin and simvastatin significantly decreased the number of stromal cells in the pancreas of male KC mice (59.4 ± 4.6% vs. 48.8 ± 14.4%; HFCD vs HFCD + Met + Sim; *p* < 0.05) (Fig. 2A, D). In contrast, the administration of metformin and simvastatin to female KC mice fed the HFCD had no significant effect on pancreatic acinar cell loss, pancreatic inflammation score, or pancreatic fibrosis compared to HFCD alone (Fig. 2A–D).

### The combination of low-dosage metformin and simvastatin attenuates pancreatic neoplasia in male KC mice fed the HFCD

**Figure 3:**
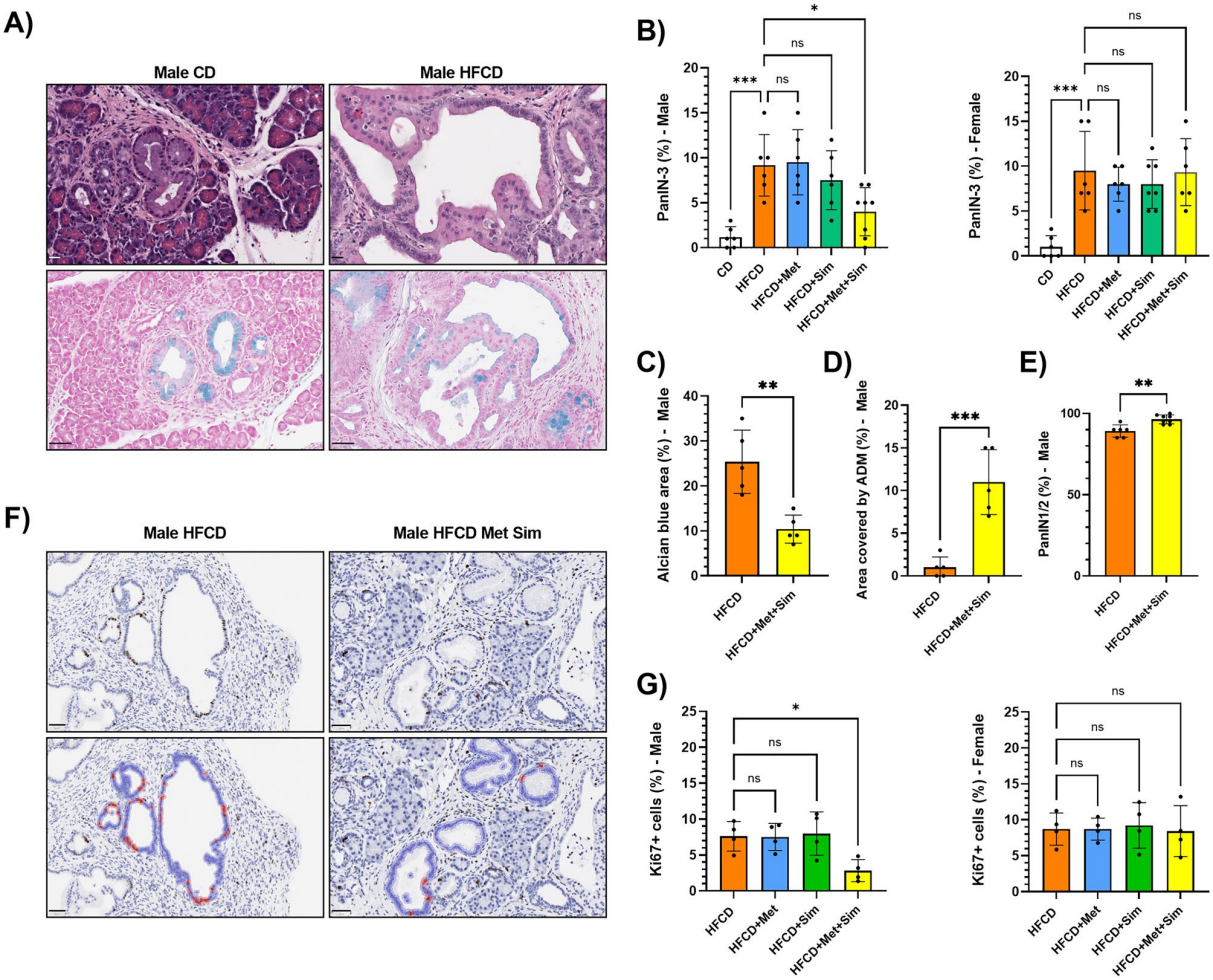
(**A**) Representative microscopy images of the pancreas stained with hematoxylin and eosin (top row; scale bar: 20 µm) or Alcian blue (bottom row; scale bar: 50 µm) of male KC mice fed the control diet (CD, left panels) or high fat, high calorie diet (HFCD, right panels). (**B)** Percentage of PanIN-3 lesions in male (left panel) and female (right panel) KC mice fed the CD, HFCD, HFCD + metformin (Met), HFCD + simvastatin (Sim), or HFCD + Met + Sim. Data are presented as scatter plots with bars (mean ± SD). ***p ≤ 0.001, *p ≤ 0.05, ns = not significant. (**C)** Percentage of the area of Alcian blue positive lesions in relation to the entire cross section of the pancreas in male KC mice fed the HFCD or HFCD + Met + Sim. Data are presented as scatter plots with bars (mean ± SD). **p ≤ 0.01. (**D)** Percentage of the area covered by ADM lesions in relation to the entire cross section of the pancreas in male KC mice fed the HFCD or HFCD + Met + Sim. Data are presented as scatter plots with bars (mean ± SD). ***p ≤ 0.001. (**E)** Percentage of low-grade PanIN-1 and -2 lesions in male KC mice fed the HFCD or HFCD + Met + Sim. Data are presented as scatter plots with bars (mean ± SD). **p ≤ 0.01. (**F)** Representative microscopy images of the pancreas stained with antibodies against Ki67 (upper row) of male KC mice fed the HFCD (left panel) or HFCD + Met + Sim (right panel) (scale bar: 50 µm). Brown staining indicates positivity for Ki67. Lower row represents QuPath images for automatic quantification of Ki67-positive PanIN cells of the same images shown in the top row. (**G)** Percentage of Ki67-positive PanIN cells in male (left panel) and female (right panel) KC mice fed the HFCD, HFCD + Met, HFCD + Sim, or HFCD + Met + Sim. Data are presented as scatter plots with bars (mean ± SD). *p ≤ 0.05, ns = not significant.

The enhanced fibro-inflammation in KC mice fed the HFCD was accompanied by an increase in advanced PanIN-3 lesions in both female and male KC mice (Fig. 3A, B). Alcian blue was used to stain the mucinous content of PanIN lesions (Fig. 3A). While most KC mice fed the CD displayed marked ADM with only very few advanced PanIN lesions, the pancreata of animals on the HFCD were characterized by increased PanIN-3 lesions scattered throughout the organ (CD vs. HFCD; 1.2 ± 1.2% vs. 9.2 ± 3.4% in male KC; *p* < 0.001 and 1.0 ± 1.3% vs. 9.5 ± 4.4% in female KC; *p* < 0.001) and the almost complete absence of ADMs (Fig. 3A, B). While treatment with either metformin or simvastatin alone did not significantly reduce the percentage of PanIN-3 lesions in female and male KC mice, the combination of metformin and simvastatin significantly decreased the number of PanIN-3 lesions in male KC mice (HFCD: 9.2 ± 3.4% vs. HFCD + Met + Sim: 4.0 ± 2.7%; p = 0.02) but not in female KC mice (Fig. 3A, B). In addition, compared to male KC mice fed the HFCD, treatment with metformin and simvastatin significantly reduced the area of Alcian blue positive lesions (as markers of PanINs) and increased the extent and number of ADM and low PanIN (PanIN-1 and -2) lesions (Fig. 3C–E).

Since our previous work showed that metformin and simvastatin as single treatments at high doses inhibited proliferation and colony formation in murine and human pancreatic cancer cells^11,12,24^, we performed Ki67 immunohistochemistry to measure the effects of metformin and simvastatin on PanIN cell proliferation. While 7.6 ± 2.0% of PanIN cells in male KC mice fed the HFCD showed positive Ki67 staining, this percentage was reduced to 2.8 ± 1.5% in male KC mice fed the HFCD plus metformin and simvastatin (p = 0.04; Fig. 3F, G). Again, the combination of metformin and simvastatin had no effect on Ki67-positivity in PanIN lesions in female KC mice compared to HFCD alone. In addition, single treatments with metformin and simvastatin did not reduce PanIN cell proliferation in male and female KC mice fed the HFCD (Fig. 3F, G).

### The combination of metformin and simvastatin at low doses attenuates YAP/TAZ transcriptional activity in male KC mice fed the HFCD

**Figure 4:**
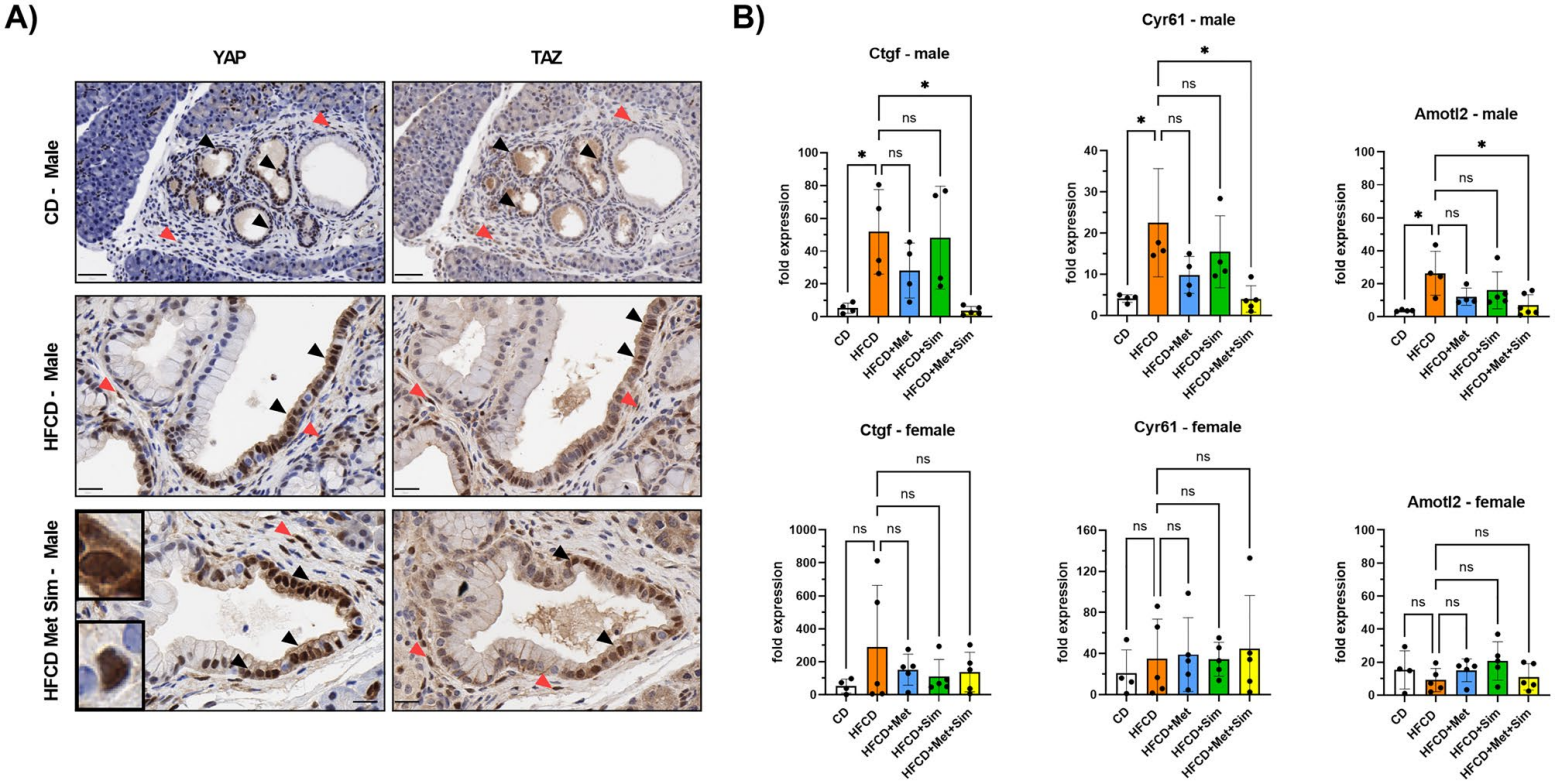
**(A)** Representative microscopy images of the pancreas stained with antibodies against YAP (left column) or TAZ (right column) of male KC mice fed the control diet (CD, top row), high fat, high calorie diet (HFCD, middle row), or HFCD + metformin (Met) + simvastatin (Sim) (bottom row). Brown staining indicates positivity for YAP or TAZ. Black arrowheads point to epithelial (PanIN, ADM) cells, and red arrowheads to stromal cells positive for YAP or TAZ (scale bar: 50 µm for top row and 20 µm for middle and bottom row). Upper inset shows a PanIN cell with nuclear and cytoplasmic YAP staining; lower inset depicts a PanIN cell with strong exclusive nuclear YAP staining. (**B)** Real-time quantitative PCR analysis of *Ctgf* (left column), *Cyr61* (middle column), and *Amotl2* (right column) mRNA expression in the pancreas of male (upper row) and female (lower row) KC mice fed the CD, HFCD, HFCD + Met, HFCD + Sim, or HFCD + Met + Sim. *p ≤ 0.05, ns = not significant.

There is evidence that the anti-neoplastic activities of metformin and statins are mediated at least in part by inhibition of YAP/TAZ^12,25,26^. Metformin can decrease YAP/TAZ activity through several AMPK-dependent mechanisms^18^. In contrast, statins block mevalonate synthesis and subsequent generation of geranyl–geranyl pyrophosphate (GG-PP), leading to decreased activation of Rho small GTPases, which are important regulators of YAP/TAZ nuclear translocation and transcriptional activity^22^ (Supplementary Figure 1). To support the hypothesis that the beneficial effects of the combination of metformin and simvastatin on pancreatic cancer development in the KC mouse model are mediated through inhibition of YAP/TAZ activity, we first examined the YAP and TAZ expression patterns in the pancreas of KC mice. Immunohistochemistry revealed that both YAP and TAZ are strongly expressed in ADM and PanIN lesions with no discernable difference between any treatment groups (Fig. 4A). The staining pattern in all groups appears heterogenous with strong nuclear staining and absence of expression sometimes within the same PanIN lesion (Fig. 4A). PanIN cells were usually either positive for both YAP and TAZ, or negative for both proteins. No correlation between the treatment and the subcellular (nuclear versus cytoplasmic) localization of YAP and TAZ could be identified. In addition to transformed pancreatic epithelial cells, a small percentage of stromal cells were also found to be positive for YAP and TAZ expression (Fig. 4A), as reported previously^27^. To investigate whether the combination of metformin and simvastatin affected YAP and TAZ transcriptional activity, we performed real-time PCR analysis of YAP and TAZ target genes using total pancreatic RNA. Compared to the CD, the mRNA expression levels of *Ctgf (CCN2)*, *Cyr61 (CCN1)*, and *Amotl2*, known products of YAP- and TAZ-associated transcriptional activity, were significantly elevated in the pancreas of male KC mice fed the HFCD (Fig. 4B). This increase in transcript levels was essentially eliminated in KC mice fed the HFCD and treated with metformin and simvastatin (Fig. 4B). Treatment with either metformin or simvastatin alone had no significant effect on *Ctgf*, *Cyr61*, and *Amotl2* mRNA levels in male KC mice, compared to HFCD. In contrast, neither the HFCD alone (compared to the CD) nor any of the treatment groups (compared to the HFCD) had any significant effect on the *Ctgf*, *Cyr61*, and *Amotl2* mRNA levels in female KC mice (Fig. 4B). Interestingly, in direct comparison, female KC mice fed the CD had about 5–10 times higher mRNA levels of all three genes in the pancreas than male KC mice, suggesting a higher baseline activation of YAP/TAZ in female mice.

### Metformin and simvastatin synergistically inhibit colony formation of murine and human pancreatic cancer cells

**Figure 5:**
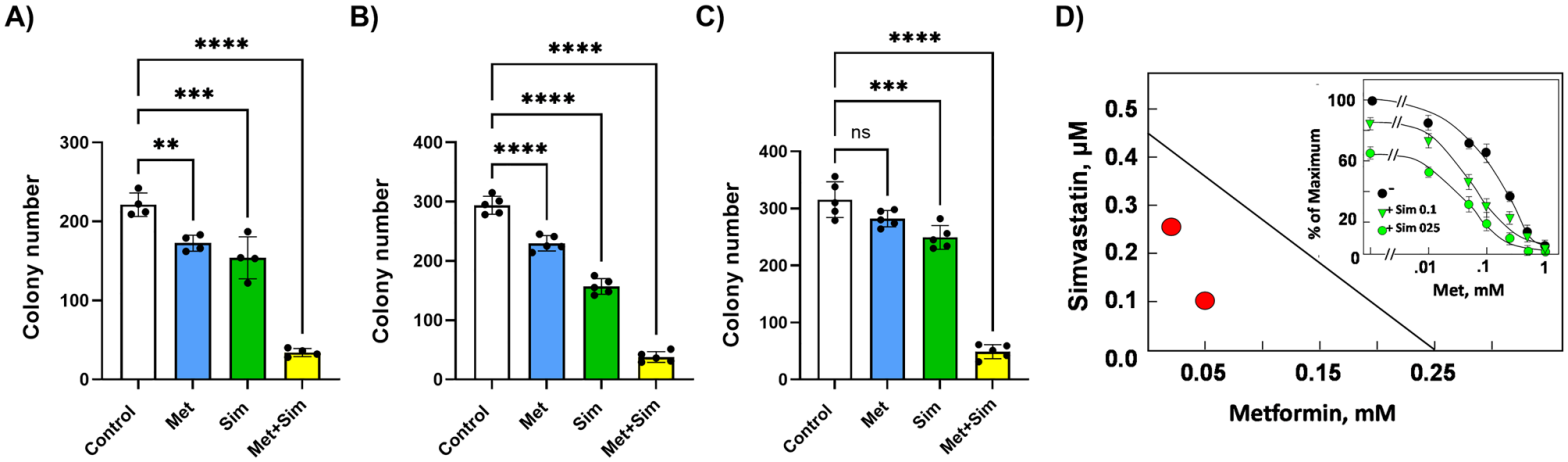
Colony formation assay of murine KC (**A**), PANC-1 (**B**), and MIA PaCa-2 (**C**) cells treated with 0.3 µM simvastatin (Sim), 0.05 mM metformin (Met), or a combination of both. Data are presented as scatter plots with bars (mean ± SD). **p ≤ 0.01, ***p ≤ 0.001, ****p ≤ 0.0001, ns = not significant. (**D)** Synergistic effects of metformin and simvastatin on inhibition of colony formation in PANC-1 cells were analyzed by isobologram analysis. The EC_50_ of simvastatin and metformin were 0.45 µM and 0.25 mM, respectively. Red circles represent the EC_50_ of metformin with simvastatin at 0.1 or 0.25 µM; their position under the isobole indicates a synergistic effect. Inset: Dose response of metformin (Met) without or with simvastatin (Sim).

Having demonstrated that metformin and simvastatin at low concentrations inhibited pancreatic cancer development in male KC mice fed the HFCD, we investigated whether metformin acts synergistically with simvastatin to inhibit pancreatic cancer cell growth in vitro. Single treatment with metformin at 0.05 mM or simvastatin at 0.3 µM significantly inhibited colony formation in murine KC cells by about 30–35% (Fig. 5A). However, the combination of metformin and simvastatin drastically reduced the number of colonies by over 80% (221 ± 15 vs. 34 ± 5, control vs. simvastatin + metformin; *p* < 0.0001). The strong inhibitory effect of the combination of metformin and simvastatin on colony formation was also seen in two human pancreatic cancer cell lines (Fig. 5B, C). In PANC-1 cells the combination of metformin and simvastatin decreased the number of colonies from 294 ± 12 to 38 ± 9 (*p* < 0.0001) and in MIA PaCa-2 cells from 315 ± 31 to 49 ± 12 (*p* < 0.0001). Isobologram analysis revealed a synergistic effect of metformin and simvastatin in inhibiting colony formation (Fig. 5D).

## Discussion

The most salient outcome of this study was the finding that the combination of low doses of metformin and simvastatin reduces pancreatic fibro-inflammation and pancreatic cancer development, as demonstrated by a decrease in PanIN-3 lesions in male KC mice with diet-induced obesity. The finding that the combination of metformin and simvastatin increased the area and number of ADM and low-grade PanIN lesions in male KC mice fed the HFCD suggests that both drugs combined inhibit or delay PanIN progression. We have previously reported that oral administrations of either metformin^11^ or simvastatin^12^ at clinically relevant but relatively high doses attenuate pancreatic neoplastic progression in KC mice fed a high fat diet. The impetus for investigating lower doses of both drugs was the known risk of clinically significant side effects of metformin and simvastatin in a subset of patients^16,17^. Since the beneficial effects of both metformin and simvastatin may be mediated by their separate but converging inhibitory action on the transcriptional activity of YAP and TAZ^22^, we hypothesized that the combination of metformin and simvastatin have a more pronounced effect than either drug alone. In fact, the doses of metformin and simvastatin used in this study (4–5 times lower than in previous studies^11,12^) were deliberately chosen to have only a very slight or no effect at all. Our results support the hypothesis, clearly demonstrating that the combination of metformin and simvastatin has a significant beneficial effect while either drug alone fails to affect pancreatic fibrosis, inflammation, and neoplastic progression in KC mice. A potential synergism between metformin and simvastatin in our animal study is supported by in vitro experiments showing that the combination of both drugs synergistically inhibits pancreatic cancer cell growth. Our finding that only the combination of metformin and simvastatin reduced YAP/TAZ transcriptional activity (as measured by quantification of target gene expression) in the pancreas of male KC mice furthermore supports the hypothesis that the synergistic effects of metformin and statins occur on the level of inhibiting YAP/TAZ activity. Synergistic effects of metformin and statins have been reported in epidemiological and clinical studies of patients with various malignancies^28,29^, as well as experimental models^30,31^. However, to our knowledge, a low dosage combination of metformin and simvastatin has not yet been investigated in experimental models of pancreatic cancer.

The mechanisms underlying the beneficial effects of metformin and simvastatin in this study may include direct inhibitory effects on pro-oncogenic signaling pathways in pancreatic cells and indirect, systemic effects. We have previously shown that metformin at lower concentrations attenuates pancreatic cancer cell proliferation through AMP-activated protein kinase (AMPK)-dependent inhibition of mammalian target of rapamycin complex 1 (mTORC1) and extracellular signal-regulated kinase (ERK) signaling^24,32^. Other groups have demonstrated that metformin downregulates the expression and activity of YAP and TAZ in various cancer models through AMPK-dependent and -independent mechanisms^26,33^. Our previous study showed that oral administration of a high dose of metformin stimulates AMPK activity (as seen by an increase in acetyl-CoA carboxylase phosphorylation), reduces the phosphorylation of mitogen-activated protein kinase kinase (MEK), ERK, and S6, and lowers the expression of YAP and TAZ in the pancreas of KC mice with diet-induced obesity^11^. In addition, we reported that simvastatin inhibits colony formation and YAP activation in pancreatic cancer cells through the reduction of mevalonate synthesis^12^ and knock down of YAP/TAZ by small interfering RNA inhibited cell proliferation and colony formation of human pancreatic cancer cells^34^.

Direct effects of metformin and simvastatin on transformed pancreatic cells in the current study are supported by the observations of decreased proliferation of PanIN cells (as measured by Ki67 staining) and the expression of YAP and TAZ, the putative mediators of metformin and simvastatin action, in pancreatic epithelial and stromal cells. We could not detect a consistent shift towards cytoplasmic localization of YAP or TAZ (indicating inhibition of transcriptional activity^35^) within PanIN cells in KC mice treated with metformin and simvastatin, which may be due, in part, to the cell context-specific, transient nature of nucleo‐cytoplasmic shuttling of YAP and TAZ^36^. However, YAP/TAZ target genes were significantly reduced in the pancreas of male KC mice treated with metformin and simvastatin compared to those fed high fat diet alone, clearly demonstrating a suppression of YAP and TAZ transcriptional activity by the combination of both drugs. Since we performed PCR analysis of the whole pancreas, we cannot identify the cell type(s) that contributed to the observed decrease in YAP and TAZ target genes. Because YAP and TAZ are also expressed in stromal cells, in agreement with previous reports^27,37^, cellular actions on pancreatic fibroblasts could also contribute to the effects of metformin and simvastatin in our study. This is corroborated by reports showing an inhibitory effect of metformin and statins on pancreatic stellate cells and the formation of desmoplasia^38–40^. As alpha smooth muscle actin-positive pancreatic stellate cells express YAP and TAZ in human and murine PDAC^27^, an inhibitory action of metformin and statins on YAP/TAZ activity, as seen in our study, can plausibly disrupt the pro-fibroinflammatory role of pancreatic stellate cells and the pro-tumorigenic crosstalk between stellate and cancer cells^37^. This idea is supported by our finding of a substantially reduced desmoplastic reaction in male KC mice fed the HFCD and treated with metformin and simvastatin.

Despite evidence of direct actions on signaling pathways in pancreatic cells, we cannot rule out that indirect, systemic effects of the combination of metformin and simvastatin (e.g. effects on insulin sensitivity, gut microbiome, or lipid metabolism^18^) also contribute to the beneficial effects on pancreatic fibro-inflammation and cancer development in our study. We have previously reported that KC mice fed a HFCD develop hyperinsulinemia^14,15^, which is normalized by oral administration of a high dose of metformin^11^. In another study, we demonstrated that oral high-dose metformin counteracts the microbial dysbiosis in the duodenum of KC mice fed a high fat diet^41^. In the current study, we could not detect any significant changes in circulating glucose and insulin levels in KC mice fed the HFCD and treated with low doses of metformin alone or in combination with simvastatin (data not shown). In addition, no differences among all groups are noted in circulating cholesterol and triglyceride levels here (data not shown) or in previous studies^14,15^. The lack of detectable effects on blood metabolic parameters in KC mice treated with metformin and simvastatin supports the notion that the beneficial effects of the combination of metformin and simvastatin may not have been mediated by indirect, systemic metabolic effects. However, since a significantly reduced weight gain was observed only in male KC mice fed the HFCD and treated with metformin and simvastatin (compared to HFCD alone), which is associated with a significant decrease in pancreatic fibro-inflammation and cancer development only in this cohort, we cannot rule out some contribution of the anti-obesity effects of the combination of metformin and simvastatin on pancreatic cancer development in this study. The reduced weight gain in male KC mice fed the HFCD and treated with metformin and simvastatin may be attributed to metformin’s anti-obesity effects, as seen in earlier studies^11^.

An interesting observation in the current study is that the beneficial effect of the combination of metformin and simvastatin on weight gain and pancreatic pathology is only seen in male, but not female KC mice. There is evidence in the literature pointing to sex differences of the effect of metformin in humans^42,43^, however, the underlying mechanisms remain obscure. Compellingly, a longitudinal study in mice showed that male C57BL/6 mice (same background strain as in our study) treated with metformin lose weight and increase their lean-to-fat ratio, while, in contrast, female mice are resistant to changes in body weight and body composition^44^. Our real-time PCR data revealed that YAP and TAZ target genes in the pancreas of KC mice fed the CD and HFCD are several-fold higher in females than in males, raising the intriguing possibility that a higher baseline activity of YAP and TAZ in female mice contributes to a “resistance” to the beneficial effects of metformin and simvastatin. Treatment with low doses of both drugs in female mice may thereby be insufficient to lower YAP and TAZ activity to a level that elicits suppressive effects on pancreatic cancer development. In a similar context, estrogens have been reported to regulate Hippo signaling via G-protein coupled estrogen receptor (GPER) in breast cancer leading to activation of YAP and TAZ^45^. Importantly, GPERs have been found to be expressed in PDAC^46^. It is plausible that higher estrogen levels in pre-menopausal female KC mice increase pancreatic YAP/TAZ expression and activity via GPER resulting in “resistance” to the inhibitory effects on YAP/TAZ activity of low doses of metformin and simvastatin in our study. However, further studies are clearly needed to investigate the exact mechanisms underlying the striking difference between female and male KC mice in response to the drug combination.

In conclusion, our study reveals beneficial effects of a combination of low-dose metformin with low-dose simvastatin on pancreatic fibro-inflammation and cancer development in male KC mice fed a HFCD. Sex differences between female and male KC mice in the response to metformin and simvastatin are observed. Our results may be of translational importance for future clinical trials testing the efficacy of metformin and simvastatin in preventing pancreatic cancer progression in humans.

## Methods

### Experimental animals

*LSL-Kras*^*G12D/*+^ and *p48-Cre*^+*/−*^ mice (rederived on a C57BL/6 background) were bred and individually tagged offspring (female and male) with the correct genotype (*Kras*^*G12D/*+^*;p48-Cre*^+*/−*^, named KC) were randomly assigned to a control diet (CD), a high fat, high calorie diet (HFCD), HFCD with metformin in drinking water (1 mg/ml), HFCD with simvastatin (50 mg/kg diet), or HFCD with simvastatin (50 mg/kg diet) and metformin in drinking water (1 mg/ml) at one month of age. All mice were socially housed and had free access to diets and water. Bodyweights were measured once per week and the general health and behavior of the animals were assessed daily. At 4 months of age, cohorts of male and female mice were sacrificed after fasting overnight, and tissues and plasma were harvested. All studies involving animals were reviewed and approved by the Chancellor’s Animal Research Committee of the University of California, Los Angeles in accordance with the NIH Guide for the Care and Use of Laboratory Animals (protocol #2011-118). No animal in this study showed signs of advanced tumor development (ascites, palpable mass, jaundice, cachexia, or weight loss of more than 20%) and needed to be prematurely euthanized. None of the animals needed analgesics during the study for pain or discomfort and died without euthanasia. All animals were euthanized using carbon dioxide inhalation (from a compressed commercial cylinder utilizing a flow meter to deliver 30–70% of the chamber volume/minute) for at least 5 min followed by a secondary method of euthanasia (thoracotomy and major organ harvest). The animal study is reported in accordance with ARRIVE guidelines.

### Genotyping analysis

*LSL-Kras*^*G12D*^ and *Cre* alleles were detected prior to randomization to the experimental cohorts by polymerase chain reaction (PCR) analysis of genomic DNA isolated from tail biopsies by Transnetyx, Inc (Cordova, TN). Mice with both *LSL-Kras*^*G12D*^ and *Cre* alleles (KC) were enrolled in the study.

### Experimental diets

All diets were prepared by Dyets, Inc. (Bethlehem, PA). A detailed composition of the diets was described previously^15^. In short, the CD contained 12% calories from fat, while 40% of calories in the HFCD stemmed from fat (corn oil-based). Simvastatin (Sigma-Aldrich, St. Louis, MO) was added to the HFCD at a concentration of 50 mg/kg diet by Dyets, Inc. All diets were stored at − 20 °C (long-term) or 4 °C (short-term) in sealed containers and prepared under low light conditions. Fresh diets were added once per week. Reverse osmosis water supplemented with metformin (Sigma-Aldrich) was made fresh and replenished weekly.

### Pancreas histology

Hematoxylin and eosin (H&E) stained tissue sections (fixed in formalin and embedded in paraffin) of the pancreas were assessed in a blinded fashion as described previously^15^. For the quantification of ADM and PanIN lesions histopathologic criteria as recommended elsewhere were used [47, 48]. Criteria for advanced murine PanIN-3 lesions included: papillary or micropapillary architecture, “budding off” of small clusters of epithelial cells into the lumen and luminal necrosis, loss of nuclear polarity, nuclear irregularities, presence of mitosis. The presence of several of these features was used for the classification of a murine PanIN-3 lesion. For each animal, approximately 100 pancreatic ducts (body and tail of the pancreas) were quantified and the proportion of PanIN-1/2 and -3 lesions to the overall number of pancreatic ducts was recorded. The area of ADMs as a percentage of the cross section of the pancreas was quantitated by QuPath^49^ (open source, version 0.2.1). Pancreatic inflammation was scored semi-quantitatively as described before^15^. Briefly, pancreatic inflammation was given an index score (0–12) reflecting the sum of individual scores (0–4) for loss of acinar parenchyma, inflammatory cell infiltration, and fibrosis. Acinar cell loss was scored based on the percentage loss across the entire cross-section, inflammatory cell infiltration was quantitated by the average number of lobular inflammatory cells per high-power field, and degree of pancreatic fibrosis was based on the cumulative area of stromal fibrosis across the entire pancreas.

For the quantification of stromal cells, H&E slides were scanned using the Aperio AT2 microscope slide scanner (Leica Biosystems, Deer Park, IL) and imported into QuPath. Each pancreas segment was captured as a single object with all cells detected. Object classification was performed with training for ~ 5–6 captures per segment to classify > 3000 stroma-associated cells and extended to the entire segment containing > 200,000 cells.

### Histological staining

Sections from formalin-fixed, paraffin-embedded tissues were deparaffinized, hydrated with distilled water, stained with hematoxylin for 8 min, washed, and immersed in Picro-sirius red (Sigma-Aldrich) for one hour. After washing, slides were dehydrated in ethanol and xylene and mounted. For alcian blue, tissue sections were deparaffinized, hydrated with distilled water, treated with 3% glacial acid solution for 3 min, and then immersed in Alcian blue solution (Sigma-Aldrich) for 30 min. Slides were counterstained in Nuclear Fast Red solution (Sigma-Aldrich) for 5 min, rinsed in distilled water, dehydrated in 95% ethanol and xylene, and then mounted. The area of Alcian blue positive lesions in relation to the entire cross section of the pancreas was quantitated by QuPath.

### Immunohistochemistry

Fully automated immunohistochemical detection was performed using the Bond RX system (Leica Biosystems) and the Bond Polymer Refine Detection kit (Leica Biosystems, Cat# DS9800). Formalin-fixed, paraffin-embedded tissue sections (pancreatic body and tail) were dewaxed with xylene and rehydrated in an ethanol series. Antigen retrieval was performed using ER1 or ER2 buffer (BOND Epitope Retrieval Solution 1 or 2, Leica Biosystems) at 100 °C for 20 min. Sections were immersed in peroxide blocking solution for 5 min and then incubated with primary antibodies against Ki67 (Cell signaling, Danvers, MA, Cat# 12202, 1:1000 dilution), YAP (Abcam, Boston, MA, Cat# ab205270, 1:1000 dilution), and TAZ/WWTR1 (Sigma-Aldrich, Cat# HPA007415, 1:200 dilution) for 60 min. After washing, the peroxidase-conjugated EnVision + System (Agilent Dako, Santa Clara, CA, Cat# K4003) was used to visualize antigen–antibody complexes. Slides were scanned using the Aperio AT2 microscope slide scanner (Leica Biosystems).

To quantitate Ki67-positive staining, the percentage of Ki67-positive cells among a minimum of 2,000 PanIN cells from at least 10 PanIN lesions per mouse was automatically detected using QuPath.

### RNA extraction and quantitative RT-PCR

Mouse pancreatic tissue was preserved in RNAlater (Sigma-Aldrich, St. Louis, MO) and later homogenized using a motorized teflon pestle and glass mortar (Glas-Col, Terre Haute, IN). Tissues were placed in the glass mortar along with 300 µL of Lysis Buffer (PureLink RNA Mini Kit, Invitrogen, Waltham, MA) and homogenized for 20–30 s on ice. RNA was then extracted from the homogenate using the PureLink RNA Mini Kit (Invitrogen) per manufacturer’s instructions. Reverse transcription to generate cDNA was done using the iScript Reverse Transcription Supermix (Bio-Rad, Hercules, CA). Following cDNA synthesis, real-time quantitative PCR was performed on a CFX Connect Real-Time System (BioRad) using the SsoAdvanced Universal SYBR Green Supermix (BioRad) and the following primers: *Ctgf* forward: TGCGAAGCTGACCTGGAGGAAA; *Ctgf* reverse: CCGCAGAACTTAGCCCTGTATG; *Cyr61* forward: GTGAAGTGCGTCCTTGTGGACA; *Cyr61* reverse: CTTGACACTGGAGCATCCTGCA; *Amotl2* forward CAGAGGGACAATGAGCGATTGC; *Amotl2* reverse*:* TCACGCTTGGAAGAGGTCCTCA. Cycling parameters were: denaturation at 95 °C for 10 min, then 44 cycles at 95 °C for 15 s, and annealing at 60 °C for 60 s. Primer sequences were from OriGene (Rockville, MD). Expression levels were normalized to 18S rRNA.

### Cell culture

Human pancreatic cancer cell lines were obtained from ATCC. PANC-1 and MIA PaCa-2 were maintained in Dulbecco’s Modified Eagle Medium supplemented with 10% fetal bovine serum (FBS). These cell lines, authenticated by ATCC by short tandem repeat analysis, were used within 15 passages and cultured for less than 6 months after recovery from frozen stocks. Primary KC cells (expressing KrasG12D) isolated from a murine pancreatic cancer developed in KC mice and validated by PCR analysis of genomic DNA, were cultured in RPMI 1640 medium with 2 mM glutamine, 100 units/mL penicillin, and 100 µg/mL streptomycin and 5% fetal bovine serum (FBS) at 37 °C in a humidified atmosphere containing 5% CO_2_.

### Colony formation assay

For cell colony formation, 500 PANC-1, MiaPaCa-2 or KC cells were plated into 35-mm tissue culture dishes in DMEM or RPMI 1640 containing 10% FBS. After 24 h of incubation at 37 °C, cultures were incubated with DMEM (5 mM glucose) medium containing 3% FBS for PANC-1 and MiaPaCa-2 cells or RPMI 1640 (5 mM glucose) medium containing 1% FBS for KC cells in the absence or presence of simvastatin or metformin, or their combination. Colonies, consisting of at least 50 cells, were stained with Giemsa. Colony numbers from at least 4 dishes per condition were determined after 8–10 days of incubation and repeated in 3 independent experiments.

### Statistical analyses

Data are presented as mean ± SD. Differences in the mean of 2 samples were analyzed by an unpaired t test. Comparisons of more than 2 groups were made by a one-way ANOVA with post hoc Tukey analysis for pairwise comparisons and comparisons vs control. An α value of 0.05 was used to determine significant differences. Data were analyzed using GraphPad Prism 9.5.1 (San Diego, CA).

### Supplementary Information

Supplementary Information.

## Data Availability

All data generated or analyzed during this study are included in this published article.
