# Supplementary material for: Low dosage combination treatment with metformin and simvastatin inhibits obesity-promoted pancreatic cancer development in male KrasG12D mice
Source: Sci Rep. 2023 Sep 26;13:16144. doi: 10.1038/s41598-023-43498-9 (PMC10522691; doi:10.1038/s41598-023-43498-9)
Supplement: Supplementary file 1 — Supplementary Information. [file 41598_2023_43498_MOESM1_ESM.pdf]

Supplementary Material to *"Low dosage combination treatment with metformin and simvastatin inhibits obesity-promoted pancreatic cancer development in male KrasG12D mice"* by Yaroslav Teper, Linda Ye, Richard T. Waldron, Aurelia Lugea, Xiaoying Sun, James Sinnett-Smith, Oscar J. Hines, Stephen J. Pandol, Enrique Rozengurt, and Guido Eibl

Supplementary Figure 1:

Model of synergistic effects of metformin and simvastatin

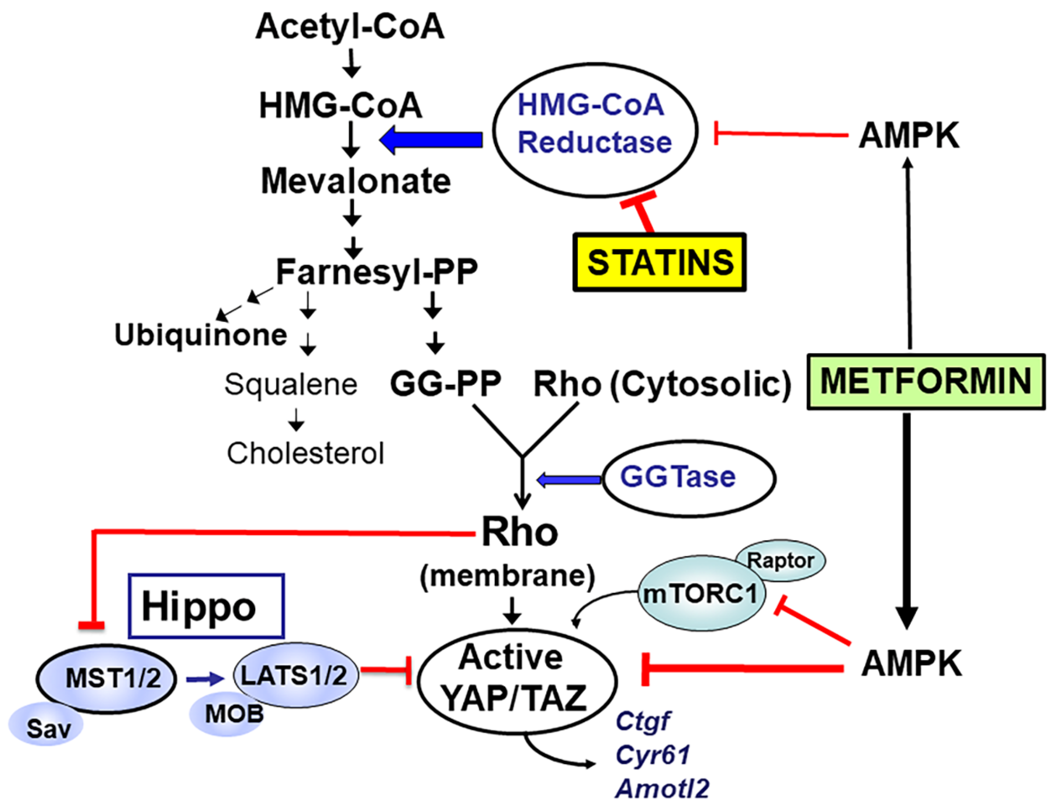

Schematic model of synergistic inhibitory effects of a combination of statins and metformin on YAP/TAZ-regulated gene expression.

Acetyl-CoA: acetyl-coenzyme A; Amotl2: Angiomotin like 2; AMPK: 5' adenosine monophosphate-activated protein kinase; Ctgf: connective tissue growth factor; Cyr61: Cysteine-rich angiogenic inducer 61; Farnesyl-PP: farnesyl-pyrophosphate; GG-PP: geranylgeranyl-pyrophosphate; GGTase: geranylgeranyl transferase; HMG-CoA: 3-hydroxy-3-methylglutaryl coenzyme A; LATS1/2: Large tumor suppressor kinase 1/2; MOB: monopolar spindle-one-binder; MST1/2: Mammalian sterile 20-like kinase 1/2; mTORC1: mammalian target of rapamycin complex 1; Sav: Salvador; TAZ: Transcriptional coactivator with PDZ-binding motif; YAP: yes-associated protein
